# Pilot study of a novel classroom designed to prevent myopia by increasing children’s exposure to outdoor light

**DOI:** 10.1371/journal.pone.0181772

**Published:** 2017-07-31

**Authors:** Zhongqiang Zhou, Tingting Chen, Mengrui Wang, Ling Jin, Yongyi Zhao, Shangji Chen, Congyao Wang, Guoshan Zhang, Qilin Wang, Qiaoming Deng, Yubo Liu, Ian G. Morgan, Mingguang He, Yizhi Liu, Nathan Congdon

**Affiliations:** 1 Henan Eye Institute, Henan Eye Hospital, Henan Provincial People's Hospital, People's Hospital of Zhengzhou University, Zhengzhou, China; 2 State Key Laboratory of Ophthalmology, Zhongshan Ophthalmic Center, Sun Yat-Sen University, Guangzhou, China; 3 The Department of Ophthalmology, The First Affiliated Hospital of Sun Yat-Sen University, Guangzhou, China; 4 School of Architecture, South China University of Technology, Guangzhou, China; 5 Guangming Eye Hospital, Yangjiang, People’s Republic of China; 6 Research School of Biology, Australian National University, Canberra, Australia; 7 Centre for Eye Research Australia, University of Melbourne, Royal Victorian Eye and Ear Hospital, Melbourne, Australia; 8 ORBIS International, New York, NY, United States of America; 9 Translational Research for Equitable Eye care, Centre for Public Health, Royal Victoria Hospital, Queen’s University Belfast, Belfast, United Kingdom; Soochow University Medical College, CHINA

## Abstract

We sought to assess light characteristics and user acceptability of a prototype Bright Classroom (BC), designed to prevent children’s myopia by exposing them to light conditions resembling the outdoors. Conditions were measured throughout the school year in the glass-constructed BC, a traditional classroom (TC) and outdoors. Teachers and children completed user questionnaires, and children rated reading comfort at different light intensities. A total of 230 children (mean age 10.2 years, 57.4% boys) and 13 teachers (36.8 years, 15.4% men) completed questionnaires. The median (Inter Quartile Range) light intensity in the BC (2,540 [1,330–4,060] lux) was greater than the TC (477 [245–738] lux, P < 0.001), though less than outdoors (19,500 [8,960–36,000] lux, P < 0.001). A prominent spectral peak at 490–560 nm was present in the BC and outdoors, but less so in the TC. Teachers and children gave higher overall ratings to the BC than TC, and light intensity in the BC in summer and on sunny days (>5,000 lux) was at the upper limit of children’s comfort for reading. In summary, light intensity in the BC exceeds TC, and is at the practical upper limit for routine use. Children and teachers prefer the BC.

## Introduction

Refractive error remains the leading cause of visual disability among children in the world today [1]. A total of 12.8 million children aged 5–15 years were visually impaired from uncorrected or inadequately corrected refractive errors in 2004, half of them dwelling in China [2]. The prevalence of myopia increases with age [3], and among secondary school children in China can reach 50–60% in rural areas [4–5] and 67.3–84.6% in urban [6–9] settings. Recent population studies have shown that only 15–20% of children who need glasses have them in urban migrant [10] and rural areas [11] of China.

The impact of uncorrected myopia on children’s well-being has been well-documented. Correction of refractive error can lead to significant improvement in educational outcomes [11], while failure to wear glasses can lead to substantial [4] and reversible [12] loss of self-reported visual function. Myopia, especially high myopia (in excess of 6D, affecting 10–20% of all children with myopia in China [13]) is associated with increased risk of retinal detachment, glaucoma and cataract [14]. Wearing spectacles is an effective treatment for refractive error, and recent trial data show that glasses are safe: their use does not worsen children’s uncorrected vision, and may even be protective compared to non-wear [15]. However, use of spectacles will not substantially reduce rates of myopia, with its associated risk of ocular pathology.

Decades of research aimed at slowing or reversing myopia progression have not yet yielded in widely-adopted interventions. Glasses and contact lenses designed specifically to reduce defocused light incident on the peripheral retina have been shown to result in modest delays in myopia progression, but high prices have limited their adoption [16–18]. Though atropine, especially in low concentrations (0.01%) has been demonstrated to slow myopia progression in children minimal deleterious effects on accommodation, pupil size or post-cessation refractive power (“rebound”), widespread uptake has been limited by lack of availability [19]. Though orthokeratology has received fairly wide acceptance in urban parts of East Asia [20], cost and concerns over infection from nocturnal use of tight contact lenses [21–22] make this approach unsuitable for large-scale programs that might significantly reduce the burden of myopia in the region.

Epidemiologic evidence suggests that increased time spent outdoors is protective against myopia in children [23]. Recent trials have shown that myopia prevalence and average refractive power are reduced in children randomized to receive additional time outdoors during the school day [24–26]. However, in view of limitations on the amount of additional daily time outdoors which parents and educational authorities will accept in China, generally an hour per day, myopia reductions have been relatively modest [24].

The mechanism for reduction in myopia risk from increased outdoor time is still not well-understood, and it has been suggested that reduced demands for near work and resulting peripheral optical defocus may be responsible [27]. However, animal studies have demonstrated reduced myopia progression with exposure to high levels of light [28–30] and wavelengths towards the blue end of the spectrum [31–32], similar to what might be encountered outdoors, though applicability of these models to human myopia is uncertain. Further, school-based surveys [23] suggest that time spent outdoors, rather than any particular activity pursued during this time, is most closely associated with reduced myopia risk. Several recent publications also suggest that light exposure in school settings may be associated with lower rates of myopia progression [33–34]. Together, these lines of inquiry suggest that exposure to higher levels of light may be the critical factor underlying protective effects of outdoor activity against myopia progression.

In the current study, we sought to examine the practicality of a novel “Bright Classroom,” designed to expose children to light levels and spectra more closely approaching those encountered outdoors, as compared to traditional classrooms. The objective was to gather quantitative data comparing light intensity, light spectrum and temperature inside and outside the Bright Classroom and in traditional classrooms, as well as subjective information from students and teachers about various aspects of their user experience in both classroom settings. The current study was neither designed nor powered to measure the impact of the Bright Classroom on progression of refractive error.

## Materials and method

The protocol for this pilot study was approved by the Institutional Review Board of the Zhongshan Ophthalmic Center (ZOC), Sun Yat-sen University (Guangzhou, China). Permission was obtained from the local Boards of Education and written informed consent was obtained from at least one parent of student participants, and from subjects themselves in the case of both students and teachers. The principles of the Declaration of Helsinki were followed throughout.

### Recruitment of subjects

A total of one out seven available fourth grade classes and two out of seven fifth grade classes at a single school were selected at random to take part in the study. Informed consent forms were distributed to all children and teachers in the selected classes. Though provisions were made for those not wishing to participate in the study to join a different class temporarily, no parents, children or teachers refused participation. In September 2014, questionnaires were administered to children and teachers asking about age, sex, wearing glasses or contact lenses and glare sensitivity. Glare sensitivity was evaluated via a five-point Likert scale from 1 (very insensitive) to 5 (very sensitive). A single Bight Classroom was constructed for the study, and participating classes utilized the classroom on a rotating basis during the entire class day (8:30–11:30 AM and 2:30–4:30 PM, with an intervening noon rest period usually spent at home) Monday through Friday for one week at a time, from September 2014 to June 2015. No classes were conducted during school vacations, on weekends or in the event of weather emergencies, when school was cancelled. Children in the final year of elementary school (Grade 6) were preparing for school-leaving examinations, and school officials requested that they not be enrolled to avoid any disruption of their studies. Children in Grades 1–3 were felt to be too young to provide reliable feedback on their user experience. Beyond membership in the selected classes and provision of informed consent, there were no additional enrollment or exclusion criteria for teachers or students to take part in the study.

### Description of the Bright Classroom

#### Local conditions

This pilot study was carried out in Yangxi county of Yangjiang city, located on the southwest coast of Guangdong Province, southern China. Yangxi county, population 463,963, had a per capita GDP of USD 6370 in 2014, among the lowest in Yangjiang. Yangjiang City, population 2,499,527, ranks in the top ten of 21 cities in Guangdong Province with a per capita GDP of USD 7250. It is situated in the tropical-subtropical transitional zone of South Asia, with an annual average temperature of 22.7°C, fluctuating throughout the year between 3.5°C and 36.3°C. Annual rainfall and sunshine duration in the area are 1680 mm and 1768 hours, respectively [35–36]. The classroom was constructed in an open area, with no direct shading from tall buildings or trees, on the grounds of the Yangxi County Experimental Primary School, located in the center of the county.

#### Configuration and materials

**Fig 1:**
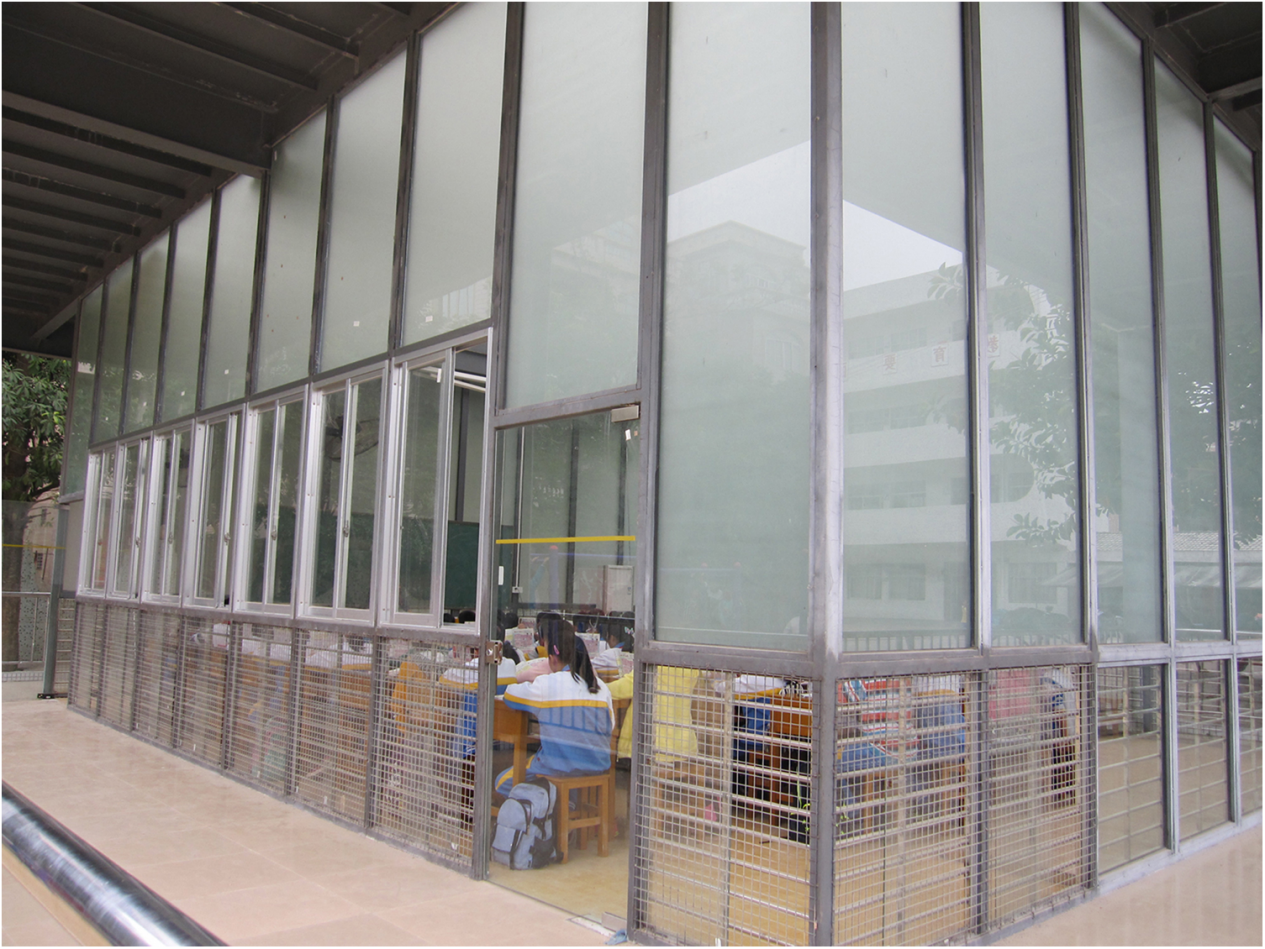
External structure of the Bright Classroom.

The Bright Classroom (Fig 1) measured 8.6 × 10.0 meters, with a height of 4.5 meters. The pillars and crossbeam were composed of steel, while the four walls and roof were made of de-polished (light-diffusing) shatterproof glass, except the bottom of each wall to a height of one meter, which was made of clear glass. The de-polished glass was used to avoid glare and visual distractions from outside of the classroom, which might interfere with teaching, while still allowing high levels of illumination internally. The clear glass allowed illumination to be further increased, while avoiding glare in the line of sight. The classroom also initially had a user-controlled shade canopy beneath the glass roof, to be deployed manually as needed in sunny conditions. To prevent flooding in the event of rain, a non-transparent overhang extending outward to a distance of 1 meter from the top of the wall was built on all 4 sides.

#### Modifications

The following modifications were made to the design in early February 2015 based on user feedback over a 6 month period from September 2014 to February 2015 (fall and winter seasons locally):

- In order to allow better temperature control inside the Bright Classroom and to increase external visibility, 14 clear glass shatterproof windows (seven on each side) on the left and right sides of the classroom were substituted for the de-polished glass. These were each 100 cm wide × 150 cm high, with a height above the ground at the bottom edge of 100 cm, and could be opened or shut manually by users.
- To improve cooling, four wall-mounted fans (FB2-40, power of each unit = 45W, Wanbao, China), two on each side, and two desktop air conditioners (KF-72LW, power of each unit = 2200W, Gree, China) were installed inside of the classroom, all of which were connected to the school electrical system.
- In view of the fact that the user-controlled canopy was kept always in the closed position, this was replaced with a fixed canopy system that could not be opened.
- An open grille was installed over the clear glass portion of the window on both the inside and outside to prevent breakage and harm to the children.

#### Cost

The total cost of building materials and construction was US$60,300, while the figure for modification and maintenance was US$2,500. Thus the cost per square meter for the Bright Classroom was $709/m^2^, compared to an average of $317/m^2^ for a conventional classroom in this region (personal report from the study architect YL, with extensive experience in constructing local school buildings).

### Data collection

#### Light intensity

We measured the light intensity inside and outside of the Bight Classroom, and in a nearby traditional classroom using an illuminometer (Z-10, Everfine Co, China), which could assess 10 points simultaneously and continuously during school days for 7–10 days in each season of the year (Autumn: 20 October to 14 November 2014; Winter: 5–23 January 2015; Spring: 8–19 April 2015; Summer: 8–19 June 2015). Measurement periods were longer prior to the modification of the classroom in February 2015, due to the need to have separate intervals of 7–10 days with the canopy deployed and retracted. All measurements were made without children in the classrooms, to avoid interfering with the equipment.

Both the Bright Classroom and traditional classroom were divided into 9 sections of equal size (each approximately 280 by 330 cm), and probes placed centrally in each section at a height of 25 cm from the desk and facing the blackboard. A single probe was placed directly outside the Bright Classroom in an area that remained unshaded throughout the day.

To explore whether light levels in the two selected traditional classrooms were representative of other classrooms in urban and rural Guangdong province, the light intensity of 29 classrooms including the two used in our study was measured between September 2015 and June 2016 at three middle schools in Guangzhou and one primary school in Yangxi. A list of classrooms was obtained for these schools. At each of the three Guangzhou schools, one building was selected at random, while all three buildings at the Yangxi school were selected. One set of classrooms from each building was chosen at random, with a single classroom located in the same position on each floor selected, so that all classrooms in a building undergoing measurement were located directly above or below one another. The indoor light intensity from the position of each desk (32–56 desks per classroom) was measured with the ceiling light turned off, using illuminometers (TA8133,TASI Electronic Co., China) with detectors oriented toward the ceiling.

#### Light spectrum

The light spectrum was measured hourly using a Spectrometer (BLACK-Comet, Stellar Net Inc., USA) continuously during school days for one week each season (measurements were carried out at the same time as assessment of light intensity, see above time schedule). Probes were placed centrally in the Bright Classroom, directly outside in an unshaded area and centrally in the traditional classroom. Separate measurements were made in the Bright Classroom with the canopy retracted and closed during the first half of the project, until a fixed canopy was installed. As above, data were collected during times when the classrooms were not in use, to avoid damage to the equipment.

#### Temperature

Three Temperature Data Loggers (Outdoors: UTBI-001, HOBO, USA; Indoors: UX100-001, HOBO, USA) were placed outdoors, in the Bright Classroom and in the Traditional Classroom. Hourly measurements were recorded continuously on school days for one school week each season (Autumn: 20–24 October 2014; Winter: 5–9 January 2015; Spring: 8–12 April 2015; Summer: 8–12 June 2015). Children were present in the classrooms during measurements.

#### Questionnaires

**Self-reported satisfaction with classrooms:** Each season, after using the Bright Classroom all day for one week, all students and teachers in each class were administered questionnaires in order to assess satisfaction with various aspects of their user experience. These had been previously created and validated by a consulting study architect (YL) as part of a doctoral dissertation (unpublished, in Chinese). The questionnaires asked about subjective assessment of brightness, glare and visibility of key classroom structures such as the blackboard and the student’s desk, as well as temperature and noise in the classroom. Identical forms were completed rating user experience of the traditional classroom, prior to using the Bright Classroom.

**Additional subjective assessment of different light levels:** In order to better understand children’s subjective response to different light levels, we designed a “Smile Thermometer” calibrated from 0 to 100. All participating children were asked to use this labeled scale to rate their comfort and ease of seeing (from 0 = Too dark to see, to 100 = Too bright to see) under classroom conditions at that moment. Children provided responses on six occasions in the Bright Classroom and once in the traditional classroom, with light intensity measured simultaneously in each case as described above.

### Statistical methods

Students' and teachers' characteristics, including age, sex, wear of glasses or contact lenses and self-reported glare sensitivity graded on a five-point Likert scale were analyzed as mean (standard deviation [SD]) for continuous variables and frequency (percentage) for categorical variables. The paired T-test was used to compare differences between the traditional and Bright Classroom in self-reported satisfaction for student data, while the Wilcoxon signed rank sum test was used for teacher data (due to non-normal distribution of the latter). A two-sided p-value< 0.05 was considered to be significant.

Linear mixed-random effect modeling was used to compare light intensity between the Bright classroom, traditional classrooms and outdoors. Log base 10 transformation was carried out on light intensity due to non-normal distribution of this variable. Two sets of analyses for self-reported satisfaction, light intensity and light spectrum were performed separately, before (combining autumn and winter data) and after (combining spring and summer data) classroom modifications in February 2015. Light spectra were compared by subjective inspection of the range of the curve from 490–560 nm, based on experimental evidence from animal studies suggesting that this part of the spectrum may be particularly important in myopia progression [31–32]. All statistical analyses were performed using a commercially available software package (Stata 13.1, StataCorp, College Station TX, USA).

## Results

Among 230 students (mean age [standard deviation, SD] 10.2 [0.75] years, 57.4% boys) participating in this pilot study, 5.24% (n = 12) wore glasses or contact lenses, while among 13 teachers (mean age 36.8 [6.34] years, 15.4% men), 46.2% (n = 6) wore them. Self-reported light-sensitivity among students (mean = 3.42 [SD = 0.95] on a 1–5 scale) was significantly higher than for teachers (1.92 [0.49], p <0.001, t test).

The Median (Inter Quartile Range, IQR) of light intensity in two traditional classrooms measured during our study, and the 27 classrooms selected from urban and rural Guangdong to provide a broader context, were 1166 (937, 2050) lux and 819 (526, 1,490) lux, respectively. The median light intensity of the former fell at the 65th percentile among the 29 measured rooms.

**Fig 2:**
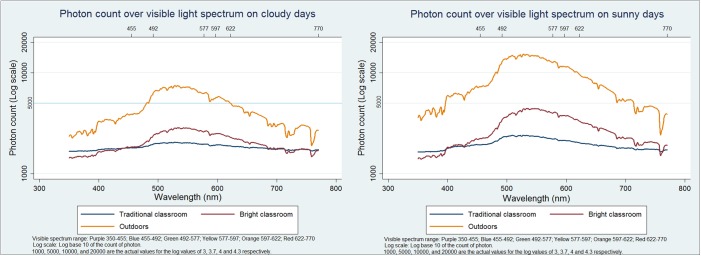
Light intensity outdoors, in the Bright Classroom and in the traditional classroom on cloudy and sunny school days in spring and summer.

The light intensity in the Bright Classroom had a median (IQR) value across all four seasons, including both sunny and cloudy days, of 2,540 (1,330–4,060) lux and a summer median of 4,220 (2,700–5,290) lux. This was greater than that in the traditional classroom (annual median [IQR] 477 [245–738] lux, P < 0.001, summer median [IQR] 610 [421–691] lux, P < 0.001), though not as high as outdoors (annual median [IQR] 19,500 [8,960–36,000] lux, P < 0.001, summer median [IQR] 20,900 [13,600–29,500] lux, P < 0.001). Fig 2 depicts light intensity in the two classrooms outdoors at different times on sunny and cloudy days in spring and summer. The relative intensity of light in the two classrooms and outdoors was similar in the autumn/winter on sunny days with the roof canopy both open and closed, prior to removal of the canopy (data not shown). The light intensity was also greater on fall/winter cloudy days in the Bright versus traditional classroom, though the difference was not significant (P = 0.056).

**Fig 3:**
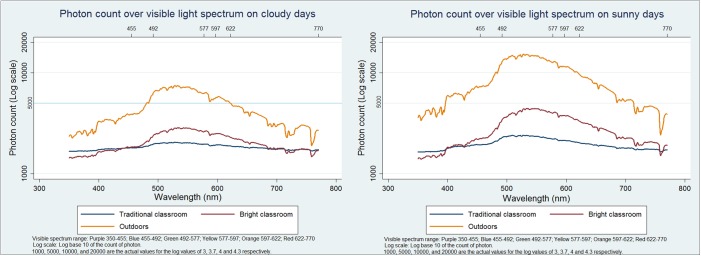
Visible light spectrum (Log scale) outdoors, in the Bright Classroom, and in the traditional classroom on cloudy and sunny school days in spring and summer.

The light spectrum in the Bright Classroom also more closely resembled that outdoors than did that of the traditional classroom on both cloudy and sunny days in both spring and summer seasons, with a more discernible peak in the range of 490–560 nm (blue-green), though this was more prominent on sunny than on cloudy days. (Fig 3) Again, the trend was similar in autumn and winter (data not shown).

**Fig 4:**
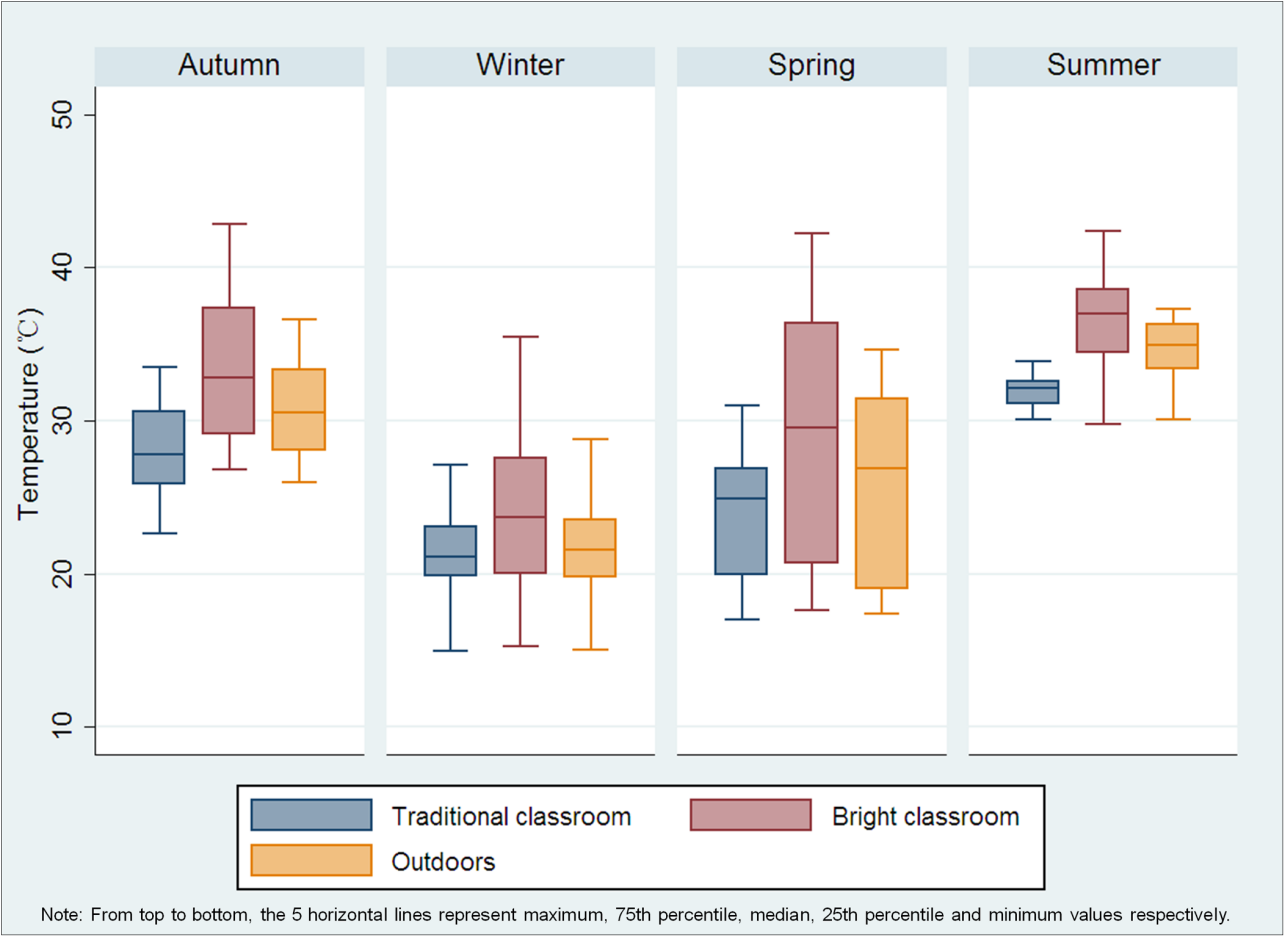
Boxplots of temperature outdoors, in the Bright Classroom and in the traditional classroom over the four seasons.

Fig 4 reveals that the temperature each season in the Bright Classroom was higher than that outdoors and in the traditional classroom, especially in summer. The mean difference ranged from 2.55 (95% Confidence Interval [CI] [1.88, 3.22], P <0.001) degrees Celsius in winter to 4.65 (95% CI [3.92, 5.38], P <0.001) degrees Celsius in summer.

Children reported their overall level of satisfaction and satisfaction with lighting in the Bright Classroom to be greater than for the traditional classroom throughout the year, both before and after the re-modeling (Table 1). Children did, however, find the Bright Classroom to be warmer and noisier than the traditional classroom, and this was true both before and after the remodeling. Table 1 gives additional sub-scores for children regarding various aspects of lighting at the blackboard, windows, children’s desks and with regard to visibility of faces and visual distractions from outside.

**Table 1:** Students' self-reported satisfaction with the traditional versus bright classroom, combining data before re-modeling, and combining data after re-modeling, based on student’s responses, (1[worst]-5[best], Mean ± SD).

Item	Combining autumn and winter data before re-modeling (N = 230)			Combining spring and summer data after re-modeling (N = 230)		
	Traditional classroom	Bright classroom	P^a^	Traditional classroom	Bright classroom	P^a^
**CLASSROOM OVERALL IMPRESSION**						
**Overall impression of the classroom**	**3.43±0.48**	**3.55±0.52**	**0.002**	**3.45±0.52**	**3.65±0.57**	**<0.001**
**WINDOWS**						
**Brightness/discomfort from direct light through windows**	3.77±0.57	3.77±0.67	0.939	**4.01±0.63**	**3.76±0.83**	**<0.001**
**CLASSROOM LIGHTING OVERALL**						
**Overall adequacy of light for vision in the classroom**	**3.83±0.63**	**3.99±0.69**	**<0.001**	**3.88±0.76**	**3.98±0.74**	**0.048**
**Overall impact of light and glare in the classroom**	**4.21±0.66**	**4.36±0.63**	**<0.001**	4.28±0.73	4.21±0.76	0.111
**Overall satisfaction with lighting in the classroom**	**3.72±0.75**	**3.86±0.81**	**0.006**	**3.84±0.78**	**3.95±0.80**	**0.032**
**BLACKBOARD**						
**Visibility of writing on the blackboard**	**4.22±0.58**	**4.33±0.54**	**<0.001**	4.28±0.60	4.32±0.57	0.210
**Brightness of light striking the blackboard**	3.79±0.42	3.81±0.47	0.513	3.77±0.49	3.82±0.49	0.178
**Impact of glare on reading words on the blackboard**	**4.34±0.51**	**4.48±0.50**	**<0.001**	4.42±0.58	4.43±0.58	0.863
**Overall satisfaction with blackboard lighting**	**3.79±0.80**	**3.94±0.76**	**0.005**	3.87±0.76	3.92±0.81	0.281
**STUDENTS’ DESKS**						
**Adequacy of light for reading at my desk**	**4.01±0.67**	**4.11±0.74**	**0.018**	**3.99±0.73**	**4.08±0.69**	**0.034**
**Brightness of light striking my desk**	3.62±0.50	3.64±0.54	0.574	**3.60±0.51**	**3.72±0.53**	**0.001**
**Impact of glare on reading material at my desk**	**4.39±0.51**	**4.49±0.52)**	**0.003**	4.44±0.58	4.39±0.60	0.079
**Overall satisfaction with lighting at my desk**	3.85±0.69	3.92±0.75	0.112	3.85±0.77	3.92±0.78	0.224
**MISCELLANEOUS LIGHTING**						
**Visibility of the teacher’s/fellow students’ faces while speaking**	**4.35±0.72**	**4.23±0.82**	**0.026**	4.24±0.86	4.29±0.83	0.375
**Distraction during class from visibility of outdoors**	4.30±0.60	4.28±0.65	0.574	**4.17±0.8**	**3.98 ±0.97**	**0.001**
**CLASSROOM TEMPERATURE/ NOISE**						
**Feel the classroom is too hot**	**3.41±1.01**	**3.00±1.00**	**<0.001**	**3.32±1.01**	**2.89±0.94**	**<0.001**
**Feel the classroom is too cold**	4.28±0.64	4.19±0.68	0.08	4.46±0.59	4.43±0.61	0.570
**Noisiness of classroom**	**2.64±0.76**	**2.82±0.90**	**0.001**	**2.48±0.86**	**2.24±0.85**	**<0.001**

**Abbreviations:** SD = Standard Deviation.

a. Paired t test for student data were used for comparing the differences between traditional classroom and open classroom.

Teachers assigned higher overall satisfaction scores to the Bright versus the traditional classroom, though the difference was statistically significant only prior to remodeling (Table 2). Teachers found the Bright Classroom significantly noisier and warmer than the traditional one, although the difference for noise was significant only after re-modeling, and for heat prior to re-modeling. Table 2 gives additional ratings from teachers for other aspects of lighting and classroom use.

**Table 2:** Teachers' self-reported satisfaction with the traditional versus bright classroom, combining data before re-modeling, and combining data after re-modeling, based on teacher’s responses, (1[worst]-5[best], Median [IQR]).

Item	Combining autumn and winter data before re-modeling (N = 13)			Combining spring and summer data after re-modeling (N = 13)		
	Traditional classroom	Bright classroom	P^a^	Traditional classroom	Bright classroom	P^a^
**CLASSROOM OVERALL IMPRESSION**						
**Overall impression of the classroom**	**3.00 (3.00–3.50)**	**4.00 (3.50–5.00)**	**0.041**	3.50 (3.00–4.00)	4.00 (3.50–4.50)	0.298
**WINDOWS**						
**Brightness/discomfort from direct light through windows**	3.00 (2.50–3.50)	3.00 (2.50–3.25)	0.398	3.00 (2.50–3.50)	3.00 (2.25–3.25)	0.278
**CLASSROOM LIGHTING OVERALL**						
**Overall adequacy of light for vision in the classroom**	3.50 (3.50–3.50)	4.00 (3.50–4.00)	0.268	3.50 (3.00–4.00)	4.00 (3.00–4.50)	0.232
**Overall impact of light and glare in the classroom**	3.50 (3.00–4.00)	3.00 (3.00–3.50)	0.221	**3.50 (2.50–4.00)**	**3.00 (2.50–3.50)**	**0.033**
**Overall satisfaction with lighting in the classroom**	3.50 (3.00–3.50)	3.00 (3.00–3.50)	0.778	3.00 (2.50–3.50)	3.00 (3.00–4.00)	0.941
**BLACKBOARD**						
**Visibility of writing on the blackboard**	**3.63 (3.50–3.75)**	**3.88 (3.75–4.38)**	**0.010**	3.88 (3.75–4.00)	4.00 (3.38–4.50)	0.806
**Brightness of light striking the blackboard**	3.75 (3.75–4.00)	3.75 (3.50–4.00)	0.317	3.50 (3.25–3.75)	3.75 (3.00–4.00)	0.158
**Impact of glare on reading words on the blackboard**	3.50 (3.25–3.75)	3.75 (3.00–4.00)	0.259	3.50 (3.25–4.00)	3.50 (3.00–4.00)	0.436
**Overall satisfaction with blackboard lighting**	3.50 (3.00–3.50)	3.00 (2.50–4.00)	0.207	3.50 (3.00–3.50)	3.50 (3.00–4.00)	1.000
**STUDENTS’ DESKS**						
**Adequacy of light for reading at my desk**	3.50 (3.00–4.00)	4.00 (3.50–4.00)	0.235	3.50 (3.00–4.00)	4.00 (3.50–4.50)	0.088
**Brightness of light striking my desk**	**3.50 (3.00–3.50)**	**3.50 (3.00–3.50)**	**0.025**	3.50 (3.50–4.00)	3.50 (3.50–4.00)	0.373
**Impact of glare on reading material at my desk**	**4.00 (3.75–4.50)**	**3.25 (3.00–4.00)**	**0.038**	3.75 (3.00–4.00)	3.50 (2.75–4.00)	0.393
**Overall satisfaction with lighting at my desk**	**3.50 (3.00–3.50)**	**3.00 (2.50–3.50)**	**0.041**	3.50 (3.00–4.00)	3.50 (2.50–4.00)	0.888
**MISCELLANEOUS LIGHTING**						
**Visibility of the teacher’s/fellow students’ faces while speaking**	4.00 (3.50–4.00)	4.00 (3.50–4.00)	0.822	3.50 (3.50–4.00)	3.50 (3.50–4.00)	0.858
**Distraction during class from visibility of outdoors**	**4.00 (3.50–4.00)**	**3.00 (2.50–3.50)**	**0.020**	3.50 (2.50–4.00)	3.00 (2.50–3.50)	0.060
**CLASSROOM TEMPERATURE/ NOISE**						
**Feel the classroom is too hot**	**3.00 (2.50–3.50)**	**2.00 (1.50–2.00)**	**0.019**	3.00 (3.00–3.00)	2.00 (1.50–3.00)	0.104
**Feel the classroom is too cold**	4.00 (4.00–4.50)	4.50 (4.50–5.00)	0.074	4.00 (3.00–4.50)	4.50 (3.00–5.00)	0.441
**Noisiness of classroom**	3.00 (2.50–3.50)	2.50 (2.00–3.00)	0.195	**3.00 (3.00–3.00)**	**3.00 (2.50–3.00)**	**0.014**

**Abbreviations:** IQR = Inter Quartile Range

a.Wilcoxon signed rank sum test for teacher data were used for comparing the differences between traditional classroom and open classroom.

**Fig 5:**
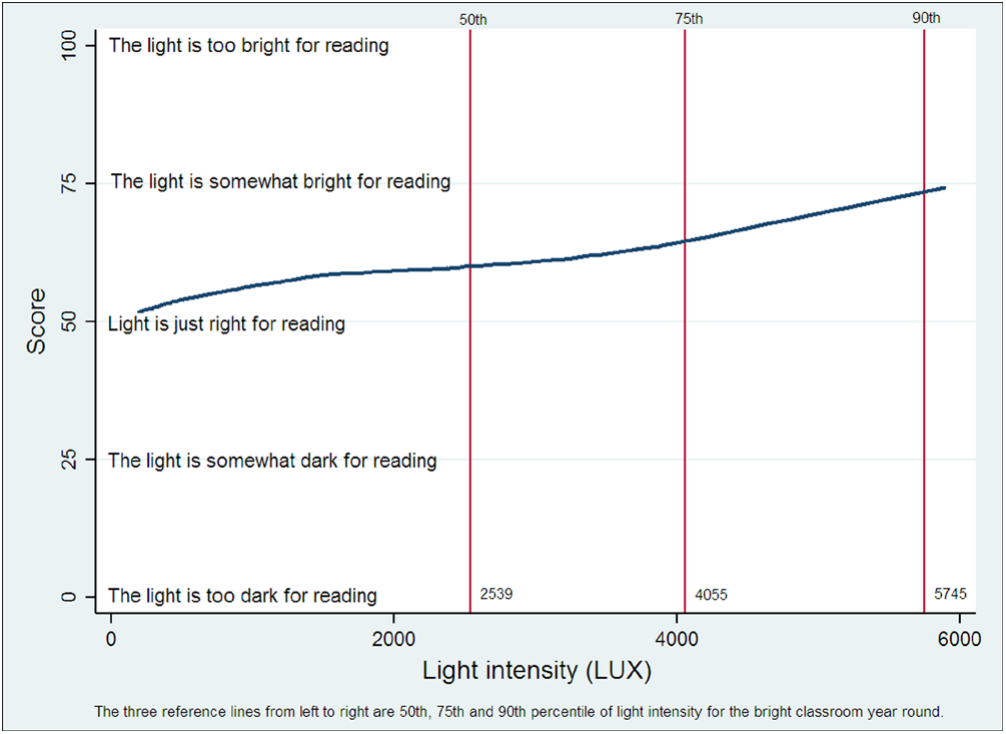
Student comfort levels at different measured light intensities.

Children’s mean comfort rating across the range of light levels normally encountered in the Bright Classroom ranged from 50 (“Light is just right for reading”) to 75 (“The light is somewhat bright for reading.”) (Fig 5). While 9.56% of children (22/230) found a light level of < 1,000 lux “Too bright,” the figure for 2,000–3,000 lux was 22.7% (50/220) and for > 4,600 lux (approaching the 90^th^ % ile value encountered during the school year, it was 31.0% (22/71). The median comfort score even at the 90^th^ % ile value was still 75 (“The light is somewhat bright for reading.”)

## Discussion

Our model Bright Classroom achieved higher overall satisfaction scores than traditional classrooms among both children and teachers, and light levels were considerably higher than in traditional classroom settings. While light intensity was lower in the Bright Classroom than outdoors, children’s feedback on reading comfort at different intensities suggested that the levels reached in the Bright Classroom may constitute a practical upper limit for comfortable learning: at the highest light intensities observed during the year, some children had already begun to report that conditions were too bright for reading. In view of evidence from animal studies that light at the blue-green segment of the spectrum may retard myopia [31–32], it was encouraging that peaks in this region were more pronounced in the Bright than the traditional classroom.

The significance of this study lies in the fact that the most carefully-done and largest randomized trial in China has suggested that practically-achievable levels of outdoor activity in China, 40 minutes/day, may be sufficient to effect only modest (23%) reductions in myopia incidence among primary school aged children, and do not show significant benefit among existing myopes [24,37]. Architectural approaches such as that outlined here may offer a practical alternative to delivering relatively high-intensity light exposures for longer periods of time, thus potentially effecting greater reductions in myopia risk. Several issues, however, remain to be addressed in future work before this potential can be realized.

In the first place, the dose-response curve for children’s light exposure and reduction in myopia risk remains largely unknown with regard both to intensity and duration. It is uncertain, for example, whether intensity must reach a threshold level before any meaningful clinical effect is achieved; animal experiments suggest the intensity necessary to retard myopia progression may be high, but relevance to human children is unknown. Our results suggest that intensity levels significantly higher than that observed in the model Bright Classroom may be problematic for sustained reading, and it is unclear that periods significantly in excess of the 40 minutes reported by He et al spent outdoors in non-educational activities will be practical in China.

The cut-off light intensity most reliably distinguishing indoor from outdoor environments is around 1000 Lux [38], and for most of the day, those in the bright classroom are well above this level. However, it should be noted that in animal experiments, light intensities of at least 10,000 for several hours a day are required for prevention [28, 39–41]. Both clinical trials and epidemiological data suggest that children who are outdoors for 2–4 hours per day may experience significant reductions in myopia risk [23–25, 42–46], but there is very little evidence on the light intensities required for protection. Depending on the time of day and location, outdoor light exposures can be a few thousand Lux to several hundred thousand Lux. However, Read et al showed that what were described as moderate (652–1019 Lux) and high (mean >1020Lux) mean daily light exposures reduced axial elongation in children by at least 50%, with only very small amounts of time spent in light intensities over 5000 Lux [34]. The lower exposures apparently required for protection in humans could be related to the particular conditions imposed in animal experiments, in which a strong stimulus for eye growth and increasing myopia is imposed constantly, whereas in children, the stimulus may be weaker and discontinuous. Overall, this evidence suggests that the light exposures achieved with the current design may well provide significant protection from myopia in children, but this needs to be established in clinical trials of the bright classroom against traditional designs, which are now being planned.

Such studies would need to address the issues of heat and noise encountered in the current model classroom, as the mean scores assigned by students for both of these areas were significantly worse for the Bright Classroom than traditional classrooms, and maximum temperatures during the summer in the Bright Classroom did occasionally exceed 40 degrees A practical approach to the heat problem would appear to be commercially-available and relatively inexpensive glass products that remain permeable to visible light while efficiently blocking heat-causing infrared wavelengths [47]. Glass providing insulation against external ambient noise is also readily available [48]. The cost per square meter of this one-off model Bright Classroom was more than twice that of conventional classrooms, but presumably much of this difference might be offset by the economy of scale inherent in building Bright Classrooms in larger numbers.

If a proof of principal can be achieved and the intensity and duration of light exposure needed to retard myopia significantly can be elucidated, a variety of simpler architectural accommodations suitable to various climates in China might be possible. Retrofitting or replacing existing classroom stock as it outdates could potentially offer a more practical solution to the current myopia epidemic than attempting to affect sustained behavior change for China’s tens of millions of children. Such a national behavior program is currently being undertaken in Taiwan, “Daily 120,” involving 2 hours per day of outdoor activity, though uptake and impact are still not well understood [49]. Such a solution does offer the opportunity to address simultaneously the current epidemic of childhood obesity in China through exercise [50], though accommodations to reduce risk of sun-induced skin damage in the higher light-intensity outdoor environment may also be needed [51–53]. Any risk of dermal and/or ocular damage [54] associated with the more modestly-elevated light intensities likely achievable through architectural designs will also need to be better understood.

In a review of articles published in English in PubMed since 1980, conducted 16 March 2016, the authors were unable to identify any other studies which have examined the practicality of architectural accommodations to increase children’s intensity of light exposure as a potential myopia preventive measure. Various researchers have assessed children’s reading speed under varying ambient light conditions [55], but generally with a view to optimizing performance, rather than exploring maximum levels consistent with subjective comfort.

Strengths of the current study include collection of a variety of relevant data (light intensity and spectrum, temperature) on an intensive basis over the length of an entire school year in a setting where myopia interventions are highly relevant; detailed assessment of multiple aspects of teachers’ and students’ subjective user responses using a validated instrument; and collection of data from a large number of children on their subjective assessment of reading comfort at the full range of light intensities encountered in this model classroom setting. Weaknesses must also be acknowledged. First, the study was not designed or powered to assess any causal association between use of the Bright Classroom and incidence or progression of myopia. Secondly, only one school in a single location in Guangdong Province was included, and the number of teachers in particular was small, so any general inferences about acceptability of the Bright Classroom in other settings must be made only with caution. Children’s self-reported assessment of the classrooms, including aspects such as noise levels, is inherently subjective, and different cohorts might have yielded different responses.

The questionnaire we used to assess satisfaction with the classrooms was designed and previously used by architects familiar with the specific visual needs of classroom users, but it had not been previously subjected to the scrutiny of peer-reviewed publication. While data under different lighting conditions on objective outcomes, such as reading speed, would have been of value, such measures would have required control over light levels to allow a large number of children to be measured under standard conditions. This was not possible under the current study design. Finally, temperature and light intensity levels might have been different in other settings with different weather and climactic conditions.

Despite its limitations, the current study suggests that architectural interventions of this sort can be acceptable to teachers and students and capable of delivering levels of light intensity significantly greater than traditional classrooms at a price that could potentially be sustainable in this setting.

## Supporting information

S1 Questionnaire Questionnaire of student in Chinese. (PDF)

S2 Questionnaire Questionnaire of teacher in Chinese. (PDF)

S3 Questionnaire Questionnaire of student in English. (PDF)

S4 Questionnaire Questionnaire of teacher in English. (PDF)
