# Supplementary material for: Pilot study of a novel classroom designed to prevent myopia by increasing children’s exposure to outdoor light
Source: PLoS One. 2017 Jul 31;12(7):e0181772. doi: 10.1371/journal.pone.0181772 (PMC5536284; doi:10.1371/journal.pone.0181772)
Supplement: S2 Questionnaire — (PDF) [file pone.0181772.s002.pdf]

教室自然光线问卷调查（教师版）

ID：______　　调查日期：______年____月____日

目前，近视眼发病率在中国青少年儿童中有不断增加的趋势，多个研究发现增加户外活动可以减少青少年儿童的近视发生和发展，这个项目建造的开放性教室旨在模拟户外环境，您将在教室里学习一周时间。本调查希望可以了解学生在使用开放性教室后的舒适度和满意度，您所填写的资料仅用于科研分析，绝对保密。回答问题时，**答出您对光线的感觉是最重要的，**没有所谓的正确答案。谢谢您的合作！

0. 您本周在什么教室上课？ [1]□ 传统教室　[2]□ 开放性教室

基本信息：

1. 您给四年级的哪个班授课?（　　）班
2. 姓名：________________
3. 性别：[1]□ 男　[2]□ 女
4. 出生日期：______年____月____日
5. 您是否佩戴框架眼镜或隐形眼镜？　[1]□ 是　[2]□ 否
6. 您是否很容易受反光的影响？

   [1]□ 非常容易　[2]□ 容易　[3]□ 一般　[4]□ 不容易

   [5]□ 非常不容易
7. 您的眼睛是否患有疾病（例如：白内障、青光眼、外伤等）：[1]□ 是　[2]□ 否
8. 您本周共在该教室里给学生上了多少节课？

   _______节
9. 请按照右图(图一)所示，指出您授课时常站的大概位置是：_______区

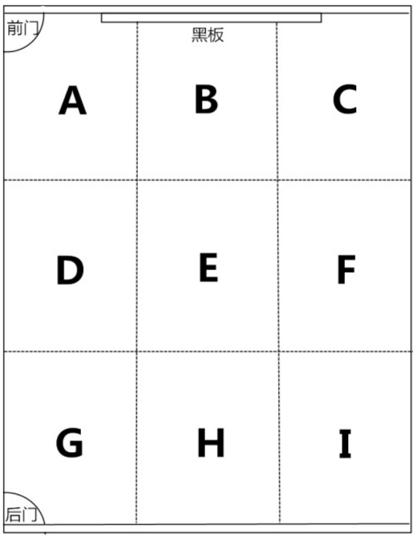

图一：查找授课时常站的位置

A. 第一部分：

当您坐在您的位置上，请描述透过窗户您能看到些什么呢？（请"√"您觉得最合适的答案）

A1. 在这一周里，您会因为从窗口透过的日光过猛而感到眼部不适吗？

[1]□ 总是会　[2]□ 经常会　[3]□ 有时会　[4]□ 很少会　[5]□ 完全不会

除此之外，还别的什么原因导致您感觉不适吗？________________________________

A2. 您觉得课室的窗户够不够大？

1□ 太大　　2□ 大了一点　　3□ 差不多　　4□ 小了一点　　5□ 太小

A3. 您觉得在该教室上课对学生上课注意力是否有影响？

1□ 严重影响　　2□ 很影响　　3□ 影响　　4□ 轻微影响　　5□ 完全不影响

B. 第二部分

当您站在授课时常站的位置，请您描述是否能看清楚黑板？（请"√"您觉得最合适的答案）

B1. 在这一周里，总的来说，在自然光下是否足够看清楚黑板上的字？

1□ 非常充足　　2□ 较充足　　3□ 差不多　　4□ 缺乏　　5□ 非常缺乏

B2. 在这一周里，总的来说，以下黑板的固定位置（如图所示三个位置）上的字是否够清楚？

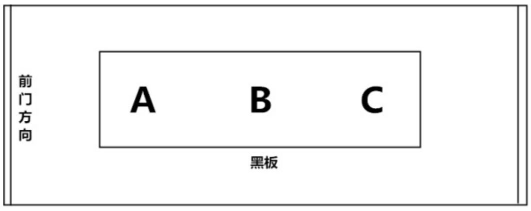

A 位置：	1□ 非常清楚	2□ 清楚	3□ 一般	4□ 不清楚	5□ 非常不清楚
B 位置：	1□ 非常清楚	2□ 清楚	3□ 一般	4□ 不清楚	5□ 非常不清楚
C 位置：	1□ 非常清楚	2□ 清楚	3□ 一般	4□ 不清楚	5□ 非常不清楚

请指出您觉得最容易看清的位置：1□ A　　2□ B　　3□ C　　4□ 都容易看清

请指出您觉得最难看清楚的位置：1□ A　　2□ B　　3□ C　　4□ 都不容易看清

B3. 在这一周里，总的来说，黑板上的明亮度如何？

1□ 太明亮　　2□ 明亮　　3□ 差不多　　4□ 暗　　5□ 太暗

B4. 在这一周里，总的来说，您觉得黑板有反光吗？

1□ 严重反光　　2□ 很反光　　3□ 反光　　4□ 稍微反光　　5□ 完全不反光

B5. 在一般情况下，这种反光会妨碍和影响您看黑板上的字吗？

1□ 严重影响　　2□ 很影响　　3□ 影响　　4□ 轻微影响　　5□ 完全不影响

B6. 当您站在讲桌讲课时，您能清楚的看清楚学生的脸吗？

1□ 非常清楚　　2□ 清楚　　3□ 一般　　4□ 稍不清楚　　5□ 非常不清楚

B7. 在这一周里，太阳光会直接照射在黑板上吗？

1□ 总是会　　2□ 经常会　　3□ 有时会　　4□ 很少会　　5□ 完全不会

B8. 总的来说，您满意课室黑板上的自然光吗？

1□ 非常满意　　2□ 满意　　3□ 一般　　4□ 不满意　　5□ 非常不满意

C. 问卷第三部分

当您需要看书或写字时，请描述讲桌上的自然光线的分布（请“√”您觉得最合适的答案）

C1. 在这一周里，总的来说，在讲桌上开展的工作（如看讲义、评阅学生的作业）是否有充足的自然光？

1□ 非常充足　2□ 充足　3□ 一般　4□ 缺乏　5□ 非常缺乏

C2. 在这一周里，总的来说，您觉得讲桌上的明亮度怎样？

1□ 太亮　2□ 亮　3□ 差不多　4□ 暗　5□ 太暗

C3. 在这一周里，总的来说，您觉得您的讲桌上有反光吗？

1□ 严重反光　2□ 很反光　3□ 反光　4□ 稍微反光　5□ 完全不反光

C4. 在一般情况下，这些反光会妨碍和影响您读书写字吗？

1□ 严重影响　2□ 很影响　3□ 影响　4□ 轻微影响　5□ 完全不影响

C5. 总的来说，您满意讲桌上的自然采光吗？

1□ 非常满意　2□ 满意　3□ 一般　4□ 不满意　5□ 非常不满意

D. 第四部分

请描述课室的自然采光情况：（请“√”您觉得最合适的答案）

D1. 在这一周里，总的来说，课室的自然采光是否充足？

1□ 非常充足　2□ 充足　3□ 一般　4□ 缺乏　5□ 非常缺乏

D2. 在这一周里，总的来说，您对课室的整体印象如何？

1□ 太过光亮　2□ 光亮　3□ 差不多　4□ 暗　5□ 太过暗

D3. 总的来说，您觉得课室里有什么地方或东西很光亮？如果有的话，是什么？

光亮的地方或东西是：____

这光亮的地方或东西对您在课堂上有什么影响？

1□ 非常分散注意力　2□很分散注意力　3□分散注意力　4□轻微分散注意力　5□完全不分散注意力

D4. 总的来说，自然光的反光对您的妨碍和影响如何？

1□ 严重影响　2□ 很影响　3□ 影响　4□ 轻微影响　5□ 完全不影响

D5. 在这一周里，太阳光会经常直接照射进课室里吗？

1□ 总是会　2□ 经常会　3□ 有时会　4□ 很少会　5□ 完全不会

D6. 当太阳光照射进课室里，会不会引起课室内过热？

1□ 总是会　2□ 经常会　3□ 有时会　4□ 很少会　5□ 完全不会

D7. 总的来说，教室内是否会让您感觉过冷？

1☐ 总是会 2☐ 经常会 3☐ 有时会 4☐ 很少会 5☐ 完全不会

D8. 上课时，您觉得课室里的噪音是怎样？

1☐ 非常吵 2☐ 吵 3☐ 一般 4☐ 安静 5☐ 非常安静

D9. 总的来说，您满意课室里的自然光线吗？

1☐ 非常满意 2☐ 满意 3☐ 一般 4☐ 不满意 5☐ 非常不满意

D10. 总的来说，你觉得课室自然光照明质量如何？

1☐ 非常好 2☐ 好 3☐ 一般 4☐ 差 5☐ 非常差

E. 第五部分

请描述课室的电灯灯光采光情况:（请“√”您觉得最合适的答案）

E1. 在开了电灯时，请您评估一下课室里的光线是否充足？

1☐ 非常充足 2☐ 充足 3☐ 一般 4☐ 缺乏 5☐ 非常缺乏

E2. 总的来说，电灯光的反光对您的妨碍或影响如何？

1☐ 严重影响 2☐ 很影响 3☐ 影响 4☐ 轻微影响 5☐ 完全不影响

E3. 在课室里，您希望是在自然光或电灯光，还是二者兼之的情况下读书？

1☐ 完全自然光 2☐ 自然光多点 3☐ 一半一半 4☐ 电灯光多点 5☐ 完全电灯光

E4. 您满意课室里的光线（自然光同电灯光一起）吗？

1☐ 非常满意 2☐ 满意 3☐ 一般 4☐ 不满意 5☐ 非常不满意

E5. 假如您对课室的光线照明有什么意见和建议，请说明如下？（您的意见将绝对保密）

____

____

再次感谢您的合作！

如果您对本次问卷有任何疑问和建议，请与中山大学中山眼科中心的康南（教授）医生 020-87682342 联系。
