# Supplementary material for: Pilot study of a novel classroom designed to prevent myopia by increasing children’s exposure to outdoor light
Source: PLoS One. 2017 Jul 31;12(7):e0181772. doi: 10.1371/journal.pone.0181772 (PMC5536284; doi:10.1371/journal.pone.0181772)
Supplement: S4 Questionnaire — (PDF) [file pone.0181772.s004.pdf]

Survey for Daylight Quality of the Open Glasses Classroom(for teacher)

ID：______ Date：______/____/____

目前，近视眼发病率在中国青少年儿童中有不断增加的趋势，多个研究发现增加户外活动可以减少青少年儿童的近视发生和发展，这个项目建造的开放性教室旨在模拟户外环境，您将在教室里学习一周时间。本调查希望可以了解学生在使用开放性教室后的舒适度和满意度，您所填写的资料仅用于科研分析，绝对保密。回答问题时，**答出您对光线的感觉是最重要的，**没有所谓的正确答案。谢谢您的合作！

Personal Information:

1. Which class do you teach? Class( ) Grade 4
2. Name：________________
3. Sex： 1☐ Boy 2☐ Girl
4. Birth Date：______年____月____日
5. Do you wear glasses or contact lenses? 1☐ Yes 2☐ No
6. Do you consider yourself as very sensitive to glare?
   1☐ always 2☐ often 3☐ sometimes
   4☐ Only occasionally 5☐ never
7. Have you had any medical problems with your eyes (e.g. cataract, glaucoma, injury, etc.)? 1☐ Yes 2☐ No
8. On average, how many classes do you have this week in this classroom? _______
9. Please look at Figure 1,roughly point out the location which you always stand during the class: Area_______

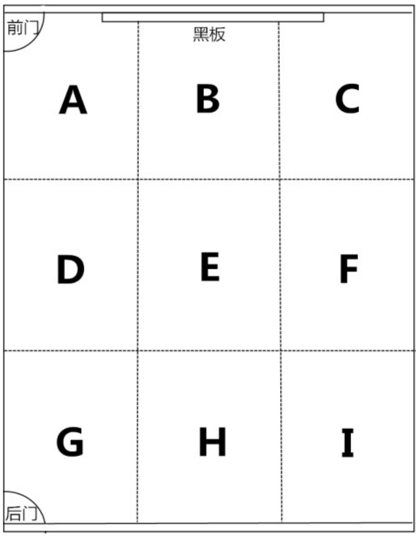

Figure1：Check out your location

A. Part One

What do you see through the windows when you sit on your seat? (Please "√" the most appropriate answer for you.)

A1. In this week, do you feel uncomfortable for the sunshine light from the windows?

1☐ always 2☐ often 3☐ sometimes 4☐ Only occasionally 5☐ never

In addition to this，is there any other reason make you feel uncomfortable?

____

A2. In your primary classroom, do you feel uncomfortable for the sunshine light from the windows?

1☐ always   2☐ often   3☐ sometimes   4☐ Only occasionally   5☐ never

A3. How do you think the size of the windows in the classroom?

1☐ too big   2☐ big   3☐ just about right   4☐ small   5☐ too small

B. Part Two

How well can you see the blackboard? (Please "√" the most appropriate answer for you.)

B1. In this week, is daylight adequate for reading on the blackboard overall?

1☐ completely adequate   2☐Fairly adequate   3☐ Just about right

4☐ fairly inadequate   5☐ Totally inadequate

B2. In this week, in general, could you clearly read what is written on the blackboard at the following different positions?

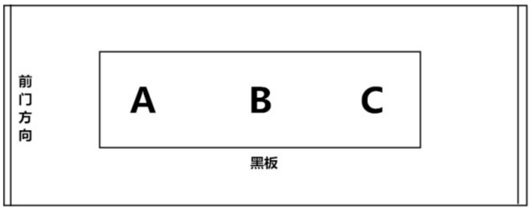

	1☐	2☐	3☐	4☐	5☐
**A:**	very clear	Fairly clear	Just about right	unclear	very unclear
**B:**	very clear	Fairly clear	Just about right	unclear	very unclear
**C:**	very clear	Fairly clear	Just about right	unclear	very unclear

Which position is the most easily for you to read? 1☐ A   2☐ B   3☐ C   4☐ all easily to read

Which position is the most difficult for you to read? 1☐ A   2☐ B   3☐ C   4☐ all difficult to read

B3. In this week, in general, how do you feel about the brightness of the blackboard?

1☐ Much too bright   2☐ too bright   3☐ just about right   4☐ too dim   5☐ much too dim

B4. In this week, in general, are there reflections on the blackboard overall?

1☐a lot of reflections   2☐many reflections   3☐some reflections 4☐a little bit reflections 5☐not at all

B5. Do the reflected glares disturb you seeing blackboard overall?

1☐ very disturbing   2☐ Fairly disturbing   3☐ disturbing   4☐ a little bit disturbing   5☐ not at all

B6. Can you see the student's face clearly when he/she is speaking to you?

1☐ very clear   2☐ Fairly clear   3☐ Just about right   4☐ unclear   5☐very unclear

B7. In this week, is there sunshine light blackboard overall?

1☐ always   2☐ often   3☐ sometimes   4☐ Only occasionally   5☐ never

B8. In general, how satisfied are you with the daylight on the blackboard?

[1]☐ very satisfied [2]☐ satisfied [3]☐ just OK [4]☐ dissatisfied [5]☐ very dissatisfied

C. Part Three

How much light reach the desk on the platform? (Please "√" the most appropriate answer for you.)

C1. In this week, is daylight adequate for reading or writing on the desk overall?

[1]☐ completely adequate [2]☐Fairly adequate [3]☐ Just about right

[4]☐ fairly inadequate [5]☐ Totally inadequate

C2. In this week, in general, how do you feel about the brightness of the desk?

[1]☐ Much too bright [2]☐ too bright [3]☐ just about right [4]☐ too dim [5]☐ much too dim

C3. In this week, in general, are there reflections on the desk overall?

[1]☐a lot of reflections [2]☐many reflections [3]☐some reflections [4]☐a little bit reflections [5]☐not at all

C4. Do the reflected glares disturb your reading or writing on the desk overall?

[1]☐ very disturbing [2]☐ Fairly disturbing [3]☐ disturbing [4]☐ a little bit disturbing [5]☐ not at all

C5. In general, how satisfied are you with the daylight on the desk?

[1]☐ very satisfied [2]☐ satisfied [3]☐ just OK [4]☐ dissatisfied [5]☐ very dissatisfied

D. Part Four

What does the classroom look like? (Please "√" the most appropriate answer for you.)

D1. In this week, in general, how do you rate the daylight level in this classroom?

[1]☐ Much too light [2]☐ too bright [3]☐ just about right [4]☐ too dim [5]☐ much too dim

D2. In this week, what is your general impression of this classroom?

[1]☐Much too bright [2]☐ too bright [3]☐ just about right [4]☐ too dim [5]☐ much too dim

D3. In general, is there anything in the classroom which is very bright and, if so, what is it?

It is :____

How do you distract by the bright thing during the class?

[1]☐ very distracting [2]☐ Fairly distracting [3]☐ distracting [4]☐ a little bit distracting [5]☐ not at all

D4. In general, is the daylight glare disturbe you?

[1]☐ very disturbing [2]☐ Fairly disturbing [3]☐ disturbing [4]☐ a little bit disturbing [5]☐ not at all

D5. In this week, is the sunshine entering the classroom most of the time?

[1]☐ always [2]☐ often [3]☐ sometimes [4]☐ Only occasionally [5]☐ never

D6. Does it ever become too hot because of the sunshine coming in through the windows?

1□ always   2□ often   3□ sometimes   4□ Only occasionally   5□ never

D7. In general, does it ever become too cold in the classroom?

1□ always   2□ often   3□ sometimes   4□ Only occasionally   5□ never

D8. During the class, are there any noises in the classroom?

1□ very noisy   2□ noisy   3□ just about right   4□ quiet   5□ very quiet

D9. In this week, how satisfied are you with the daylight in this classroom overall?

1□ very satisfied   2□ satisfied   3□ just OK   4□ dissatisfied   5□ very dissatisfied

D10. In this week, how would you describe the daylight quality in your classroom overall?

1□ Excellent   2□ good   3□ just OK   4□ poor   5□ very poor

E. Part Five

How do you feel the artificial light in the classroom? (Please "√" the most appropriate answer for you.)

E1. In general, how do you rate the lighting level, artificial light and daylight combined?

1□ Much too light   2□ too bright   3□ just about right   4□ too dim   5□ much too dim

E2. In general, does the artificial light ever cause glare strong enough to disturb you?

1□ very disturbing   2□ Fairly disturbing   3□ disturbing   4□ a little bit disturbing   5□ not at all

E3. Do you prefer studying in daylight, artificial light or a combination of daylight and artificial light?

1□ daylight only   2□ more daylight   3□ half   4□ more artificial light   5□ Artificial light only

E4. Overall, how satisfied are you with the lighting(artificial light and daylight combined) in the classroom.

1□ very satisfied   2□ satisfied   3□ just OK   4□ dissatisfied   5□ very dissatisfied

E5. If you have any opinions or comments about the lighting quality in the classroom that you like to be recorded, please write them in the space below(Your comments will be keep confidential).

____

____

____

再次感谢您的合作！

如果您对本次问卷有任何疑问和建议，请与中山大学中山眼科中心的康南（教授）医生 020-87682342 联系。
